# Carbonic Anhydrases: New Perspectives on Protein Functional Role and Inhibition in *Helicobacter pylori*

**DOI:** 10.3389/fmicb.2021.629163

**Published:** 2021-03-19

**Authors:** Cristina Campestre, Viviana De Luca, Simone Carradori, Rossella Grande, Vincenzo Carginale, Andrea Scaloni, Claudiu T. Supuran, Clemente Capasso

**Affiliations:** ^1^Department of Pharmacy, “G. d’Annunzio” University of Chieti-Pescara, Chieti, Italy; ^2^Department of Biology, Agriculture and Food Sciences, National Research Council (CNR), Institute of Biosciences and Bioresources, Naples, Italy; ^3^Proteomics and Mass Spectrometry Laboratory, Institute for the Animal Production System in the Mediterranean Environment, National Research Council (ISPAAM-CNR), Naples, Italy; ^4^Section of Pharmaceutical and Nutraceutical Sciences, Polo Scientifico, Department of NEUROFARBA, University of Florence, Sesto Fiorentino, Italy

**Keywords:** carbonic anhydrase, sulfonamide inhibitors, antibacterials, *Helicobacter pylori*, pathogens, membrane vesicles, biofilm, microbiota

## Abstract

Our understanding of the function of bacterial carbonic anhydrases (CAs, EC 4.2.1.1) has increased significantly in the last years. CAs are metalloenzymes able to modulate CO_2_, HCO_3_^–^ and H^+^ concentration through their crucial role in catalysis of reversible CO_2_ hydration (CO_2_ + H_2_O ⇄ HCO_3_^–^ + H^+^). In all living organisms, CA activity is linked to physiological processes, such as those related to the transport and supply of CO_2_ or HCO_3_^–^, pH homeostasis, secretion of electrolytes, biosynthetic processes and photosynthesis. These important processes cannot be ensured by the very low rate of the non-catalyzed reaction of CO_2_ hydration. It has been recently shown that CAs are important biomolecules for many bacteria involved in human infections, such as *Vibrio cholerae*, *Brucella suis*, *Salmonella enterica*, *Pseudomonas aeruginosa*, and *Helicobacter pylori*. In these species, CA activity promotes microorganism growth and adaptation in the host, or modulates bacterial toxin production and virulence. In this review, recent literature in this research field and some of the above-mentioned issues are discussed, namely: (*i*) the implication of CAs from bacterial pathogens in determining the microorganism growth and virulence; (*ii*) the druggability of these enzymes using classical CA inhibitors (CAIs) of the sulfonamide-type as examples; (*iii*) the role played by *Helicobacter pylori* CAs in the acid tolerance/adaptation of the microbe within the human abdomen; (*iv*) the role of CAs played in the outer membrane vesicles spawned by *H. pylori* in its planktonic and biofilm phenotypes; (*v*) the possibility of using *H. pylori* CAIs in combination with probiotic strains as a novel anti-ulcer treatment approach. The latter approach may represent an innovative and successful strategy to fight gastric infections in the era of increasing resistance of pathogenic bacteria to classical antibiotics.

## Introduction

### The Phenomenon of Antibiotic Resistance

Bacteria are unicellular organisms having a simple circular DNA as genetic material, which ensures organism reproduction (Wang and Levin, 2009). Bacterial DNA is subjected to mutations or can acquire exogenous genes from other bacteria (Watford and Warrington, 2020). In the latter context, horizontal gene transfer is generally accomplished through the transfer of a plasmid, i.e., a small circular double-stranded extrachromosomal DNA containing one or more genes, or by the fusion of extracellular membrane vesicles (MVs), which are bilayer structures produced in a budding manner by other bacteria (Grull et al., 2018; Watford and Warrington, 2020). DNA mutations, gene transfer processes as well as other mechanisms, such as changes in the outer membrane permeability, drug extrusion by efflux pumps and modification of the drug target, can induce the bacteria to develop antibiotic resistance, which is now a severe global health problem (Annunziato, 2019; Carradori et al., 2020; Watford and Warrington, 2020). Different contexts determine antibiotic resistance, namely the abuse and over-prescription of drugs recommended to treat human infections, the frequent use of such drugs in livestock farming, and the consumption of vegetables, which may be contaminated with antibiotic-resistant bacteria coming from the manure used to fertilize vegetable farming (Ahl and Buntain, 1997; Roe and Pillai, 2003; Doyle, 2015). Infections caused by resistant bacteria are treated by administering other antibiotics to which they may still be sensitive (Fernando et al., 2017). However, bacteria may acquire resistance to such new classes of antibiotics, becoming multi-resistant organisms; accordingly, it is necessary to discover novel types of antibiotics, which can overcome the pan-resistance in these microorganisms (Walsh, 2005; Collignon, 2015; Molchanova et al., 2017). Nowadays, pan-resistant infections have become an odd reality, and clinicians face this increasing problem with treating multidrug-resistant strains of many pathogens (Walsh, 2005; Molchanova et al., 2017). In Europe, it has been estimated that the resistance to first-line of antibiotics (those of first use for the treatment of infections) will remain substantially stable in 2030 compared to 2005 levels. On the other hand, resistance to second-line antibiotics used when the first-line antibiotics are ineffective, such as the third generation cephalosporins and fluoroquinolones, is expected to increase by 75% in the same period (Rossi and Sternon, 2001). For third-line antibiotics (those of the last resort, such as polymyxins), resistance is expected to double compared to 2005 levels (Hartel et al., 2016). Therefore, the super-bacteria tsunami slowly but surely is hitting, and an effective strategy is needed to counteract it. A fundamental approach is to invest in developing new drugs, and replacing those that have lost effectiveness in the therapeutic settings (Dahle and Petersen, 2013; Littman and Halil, 2016; Cheesman et al., 2017). However, the research and development of novel medicines take many years, in some cases even more than a dozen per molecule in the early stages of development, before a new product can reach the market (Littman and Halil, 2016). Consequently, it is essential to invest in a public health strategy to counteract the spread of antibiotic-resistant bacterial infections.

### The Drug Target Approach

The rapid progress in microbial genome sequencing has provided important clues to the identification of bacterial virulence factors, host specificity mechanisms, drug resistance phenomena, and genes encoding for microbial enzymes indispensable for corresponding metabolism (Selzer et al., 2000; Asif, 2012). Enzymes represent significant druggable targets since they are involved in decisive reaction catalyzing bacterial metabolic pathways, and thus are fundamental for microbe strength and virulence (Manchado et al., 2016; Sosa et al., 2018). In this context, the drug-approach method consists in: (i) the identification of essential metabolic pathways for pathogen life; (ii) the discovery of critical enzymes that are indispensable for bacterial catabolism and/or anabolism processes; (iii) the discovery of small molecules and/or peptides able interfering *in vitro* and *in vivo* with the activity of the target enzyme, and ultimately with microbial growth (Manchado et al., 2016). In general, the condition of identifying target enzymes that are present only in the microbes and not in the host is optimal for pharmaceutical purposes; for example, this situation occurs in the case of natural/synthetic molecules interfering with biosynthesis of bacterial peptidoglycan structure, which is absent in mammalian cells. However, this condition is uncommon due to the general conservation of most important metabolic pathways in all organisms. In the latter case, this limitation is overcome by designing/isolating molecules selectively inhibiting bacterial enzymes and not host protein homologs. For example, trimethoprim was found to selectively inhibit the bacterial enzyme dihydrofolate reductase (DHFR), which is ubiquitously expressed in all living organisms, but not human DHFR (Capasso and Supuran, 2014). Using NADPH as an electron donor, DHFR reduces the dihydrofolic acid (DHF) to tetrahydrofolic acid (THF). THF is the cofactor of several reactions concerning the synthesis of amino acids and nucleic acids (i.e., purines, thymidylate, methionine, glycine, pantothenic acid, and *N*-formyl-methionyl tRNA) (Capasso and Supuran, 2014). Besides, the amino acid sequence of bacterial DHFR reveals 30% of identity when compared with the human protein, and this phenomenon was associated with drug selectivity.

## Bacterial Carbonic Anhydrases as Druggable Targets

The genome exploration of microorganisms causing mammalian and non-mammalian infections as well as the genome of those considered not harmful evidenced genes encoding for an exciting class of enzymes that are involved in the metabolic balance of the bacterial carbon dioxide (CO_2_), bicarbonate (HCO_3_^–^), and protons (H^+^) (Annunziato et al., 2016; Capasso and Supuran, 2016; Del Prete et al., 2016a, b; Ozensoy Guler et al., 2016), namely carbonic anhydrases (CAs, EC 4.2.1.1). They belong to a superfamily of metalloenzymes that catalyze the physiologically crucial reversible reaction of CO_2_ hydration to HCO_3_^–^ and H^+^, according to the following chemical reaction (Capasso and Supuran, 2015a):

CO+2HO2⇋HCO+3-H+

Until now, eight CA classes indicated with α, β, γ, δ, ζ, η, θ, and ι have been described in all kingdoms of living organisms (Supuran and Capasso, 2017). All CA classes strictly conserve the CO_2_ hydration and HCO_3_^–^ dehydration mechanisms, showing an evident phenomenon of convergent evolution, having a very low sequence similarity, and different 3D molecular folds and structures (Supuran and Capasso, 2017). In Bacteria, four CA-classes (α, β, γ, and ι) regulate the CO_2_ and HCO_3_^–^ balance, being the only CA classes encoded by the bacterial genome (Capasso and Supuran, 2013, 2015b,c; Supuran and Capasso, 2015; Del Prete et al., 2020b). For enzyme catalysis, most of these CAs need Zn^2+^ as ion cofactor, which is coordinated by three amino acid residues from the protein backbone (Buzas and Supuran, 2016; Supuran, 2016e). The fourth metal ion ligand is a water molecule/hydroxide ion acting as the nucleophile in enzyme catalytic cycle (Carta et al., 2014). In particular, γ-CAs are Fe^2+^-dependent enzymes, but they are also active with bound Zn^2+^ or Co^2+^ ions; the last identified ι-CA class from the marine diatom *Thalassiosira pseudonana* prefers Mn^2+^ to Zn^2+^ as the ion cofactor. α-CAs are usually active as monomers or dimers; β-CAs are active only as dimers, tetramers, or octamers. The γ-CAs must be trimers for accomplishing their catalytic function (Di Fiore et al., 2013; De Simone et al., 2015; Ferraroni et al., 2015; Lomelino et al., 2016a). γ-CA monomers are characterized by a tandemly-repeated hexapeptide crucial for the left-hand fold of the trimeric β-helix structures (Fu et al., 2008). The X-ray structure of ι-CAs is not available at this moment. Intriguing, α- and ι-CAs catalyze also ester/thioester reactions (Supuran, 2016c; Jensen et al., 2019).

### Role of Bacterial CAs and Their Relationship With the Bacterial Lifecycle

At a physiological pH value, the naturally occurring CO_2_ hydration reaction is too slow, with a first-order rate constant of 0.15 s^–1^, while a rate constant of 50 s^–1^ was shown by the reverse reaction (Supuran and Capasso, 2017). Thus, the CA activity is connected to a very rapid process, such as that related to the transport and supply of CO_2_ or HCO_3_^–^, which is generally essential for a number of physiological mechanisms, such as pH homeostasis, secretion of electrolytes, biosynthetic processes, photosynthesis, and others (Supuran and Capasso, 2018, 2020). These processes may not be supported by the uncatalyzed reversible CO_2_ hydration reaction characterized, as noted above, by very low catalytic constants.

The presence of multiple CA genes supports the crucial role of these enzymes in prokaryotic physiology. In Gram-negative bacteria, we initially proposed that α-CAs, which are typified by a signal peptide at the *N*-terminus of the polypeptide chain, occur in the periplasmic space where they convert the CO_2_ to bicarbonate that diffuses in this environment, ensuring the microbe lifecycle (Capasso and Supuran, 2015a, 2016). In contrast, the β- or γ-classes are localized into the cytoplasm, accomplishing various intracellular functions, such as CO_2_/HCO_3_^–^ transport, pH balancing, and other (Supuran and Capasso, 2016, 2020). Recently, the existence of a short putative signal peptide at the protein *N*-terminus of some β- and γ-CAs from Gram-negative bacteria was also demonstrated; similarly, it was observed that ι-CAs in the Gram-negative bacterium *Burkholderia territorii* also present a signal peptide (Del Prete et al., 2020a). Whenever characterized by a signal peptide, β-, γ-, and ι-CAs might thus localized in the periplasmic space, having a function similar to that performed by α-CAs. Finally, taking advantage of protonography and mass spectrometry, members of α-CA class were also ascertained to occur in the outer membrane vesicles (OMVs) generated *H. pylori* strains in the planktonic and biofilm phenotypes (Ronci et al., 2019), underlying the existence of additional secretion mechanisms for these enzymes.

By affecting CO_2_/HCO_3_^–^ balance, it was demonstrated that CA activity influences a number of pivotal bacterial processes. For example, it was verified that the deletion of the gene encoding for the β-CA in *Ralstonia eutropha* is associated with an heterotrophic growth of the bacterial mutant only when elevated CO_2_ concentrations occur (Kusian et al., 2002). In *Escherichia coli*, β-CA (CynT) catalyzes the hydration of CO_2_ generated by cyanase and generates HCO_3_^–^, thus preventing final HCO_3_^–^ depletion in bacteria resulting from degradation of cyanate and/or other metabolic processes. Besides, a second β-CA (CynT2) was discovered in *E. coli*, which was demonstrated being essential for the microorganism growth at atmospheric CO_2_ (Cronk et al., 2001; Merlin et al., 2003). Finally, bacteria belonging to the genera *Buchnera* and *Rickettsia* were demonstrated being adapted to live only in niches characterized by high CO_2_ levels, and this adaptation is generally accompanied by loss of genes encoding for CAs (Ueda et al., 2012).

On the other hand, a number of examples are present in the literature concerning the relationship between CA activity and survival, pathogenicity, and virulence of several human pathogenic species. For example, the genome of *V. cholerae*, the Gram-negative bacterium responsible for cholera, was shown to encode for CAs of the β-, and γ-type, which are all involved in the production of sodium bicarbonate, a potent inducer of the cholera toxin (Abuaita and Withey, 2009). Similarly, two β-CA from *B. suis*, a Gram-negative coccobacillus responsible for brucellosis, and three β-CAs (mtCA1, mtCA2 and mtCA3) from *M. tuberculosis* (Nishimori et al., 2010), the causative agent of tuberculosis, were demonstrated being essential for the growth of the corresponding microbes (Carta et al., 2009; Ceruso et al., 2014; Singh and Supuran, 2014; Kohler et al., 2017). Analogously, the genome of *S. enterica* serovar Typhimurium, a Gram-negative bacterium causing gastroenteritis (Rollenhagen and Bumann, 2006), also encodes for a β-CA (Nishimori et al., 2011; Vullo et al., 2011) that is highly expressed during the bacterial infection, as demonstrated by *in vivo* gene expression studies (Rollenhagen and Bumann, 2006). In *P. aeruginosa* (psCA1), a Gram-negative bacterium commonly found in the environment, the β-CA gene’s deletion provoked a reduction of calcium salt depositions, impairing the microbe virulence (Lotlikar et al., 2019). Finally, it was demonstrated that various CAs encoded by the *H. pylori* genome are essential for the acid tolerance/adaptation of the microbe in the stomach, a harsh environment with pH values as low as 1–2 (Buzas, 2010; Modak et al., 2019).

### Inhibition of Bacterial CAs

With their activity, CAs continually provide the indispensable CO_2_ and HCO_3_^–^/protons to microbial biosynthetic pathways. Thus, it is immediately apparent that their inhibition might impair the survival of pathogens. CA inhibitors (CAIs) belonging to many chemical classes are known and a description is reported below.

#### Substituted Benzene-Sulfonamides and Clinically Licensed Drugs

**FIGURE 1:**
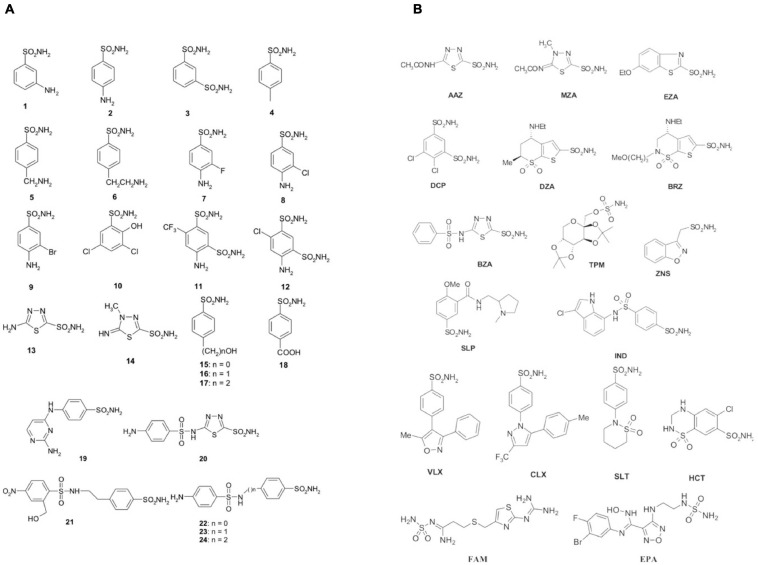
Sulfonamides and their isostere classes (sulfamates and sulfamides) as CAIs. Simple aromatic/heterocyclic derivatives **1–24 (A)**; clinically used drugs or agents in clinical development **(B)**.

The initial antimicrobial products commonly used in healthcare environments were the sulfonamides discovered by Domagk in 1935 (Otten, 1986). Prontosil was the first sulfonamide to demonstrate an intense antibacterial activity. It is a sulfanilamide prodrug, which is isosteric/isostructural with *p*-aminobenzoic acid (PABA), the substrate of dihydropteroate synthase (DHPS) (Achari et al., 1997). DHPS is a critical enzyme for folate synthesis, an essential vitamin/nutrient that mammals get from their diet. Differently from mammals, bacteria use DHPS to synthesize folate through the chemical reaction among DHPP and PABA. After sulfanilamide was demonstrated to be an effective antibacterial agent, many analogs (the sulfa drugs) entered clinical use. Today, these compounds are still used although knowing drug resistance issues. DHPS, as mentioned above, is the target of the sulfa drugs, which work because they fit into the DHPS active site and take PABA’s place. Several DHPS mutations are responsible for sulfonamide resistance (Capasso and Supuran, 2014). Sulfa drugs are derived from sulfonamides, and the presence of primary sulfonamide moieties in sulfanilamide characterizes most of the investigated CA inhibitors (CAIs) (Supuran, 2016a, b, 2017a). Sulfonamides and their structurally related derivatives, such as sulfamates and sulfamides, have the general formula A-SO_2_NH-R, where A can be an aromatic, heterocyclic, aliphatic, or sugar scaffold, while R may be hydrogen (primary sulfonamides/sulfamates/sulfamides), or a multiplicity of moieties incorporating heteroatoms (-OH, -NH_2_, etc.), as well as organic scaffolds like those said for A. Thus, a range of compounds containing the -SO_2_NH_2_ group were investigated as CAIs against bacterial CAs or CAs from other organisms (Supuran, 2017b). Figure 1 shows some of these sulfonamide inhibitors (simple derivatives **1–24** and clinically used drugs or agents in clinical development) (Carta et al., 2009; Nishimori et al., 2010, 2014; Vullo et al., 2013, 2015a,b,c; Alafeefy et al., 2015a,b; Dedeoglu et al., 2015; Abdel Gawad et al., 2016; Del Prete et al., 2016b, c, d; Diaz et al., 2016; Supuran, 2016d). Acetazolamide (**AAZ**), methazolamide (**MZA**), ethoxzolamide (**EZA**) and dichlorophenamide (**DCP**) are systemically acting antiglaucoma CAIs. Dorzolamide (**DZA**) and brinzolamide (**BRZ**) are antiglaucoma agents that function topically; benzolamide (**BZA**) is an orphan drug of this pharmacological class. Some of these compounds, such as topiramate (**TPM**), sulthiame (**SLT**), and zonisamide (**ZNS**), are antiepileptic drugs in clinical use for several decades. Other sulfonamides, such as the clinically used sulpiride (**SLP**) and the antitumor agent indisulam (**IND**), no longer in clinical development, along with the sulfonamides originally developed as COX-2 selective inhibitors [celecoxib (**CLX**) and valdecoxib (**VLX**)] were also included in our experiments. Other investigated compounds as CAIs are saccharin (**SAC**), hydrochlorothiazide (**HCT**), a thiazide diuretic (Supuran, 2008), famotidine (**FAM**), a histamine H_2_-receptor antagonist (Nguyen et al., 2020), as well as the experimental agent epacadostat (**EPA**), which acts as an inhibitor indoleamine 2,3-dioxygenase-1 (IDO1), a heme-containing enzyme (Komiya and Huang, 2018). All of them were shown to also act as CAIs primary sulfonamides as these ones inhibit CAs by binding to the Zn^2+^ ion from the enzyme active site, in a tetrahedral geometry of the metal, whereas the sulfonamide is deprotonated at the SO_2_NH_2_ moiety. The nitrogen atom of the SO_2_NH^–^ group then coordinates the Zn^2+^ ion, and participates to a network of H-bonds, which involve conserved amino acid residues (Thr199 and Glu106), which in this way anchor the inhibitor molecule to the enzyme very strongly. This has been demonstrated by X-ray crystallographic studies of many adducts of such sulfonamides with various CA isoforms. The scaffold of the inhibitor (aromatic/heterocyclic moiety) also interacts with amino acid residues from the active site, either in the hydrophilic or within the hydrophobic part of the catalytic cleft.

#### Inorganic Metal-Complexing Anions

Anions or complex molecules (such as carboxylates) can bind CAs (De Simone and Supuran, 2012). Anions may bind either to the metal ion in the tetrahedral geometry or as trigonal–bipyramidal adducts. Anion inhibitors are generally millimolar or submillimolar CAIs; they are thus less effective than sulfonamides, which may show K_Is_ in the submicromolar to the nanomolar range. However, the anion inhibition profile is essential for the comprehension of the cellular physiological processes, which see involved the CAs, as well as for the production of new forms of selective and efficient inhibitors; the latter ones may be useful in the treatment of disease caused by an alteration in the CA activity.

#### Dithiocarbamates

Other CAIs investigated as antibacterials are made of dithiocarbamates (DTCs) (Scozzafava et al., 2000, 2001; Carta et al., 2012a, b; Monti et al., 2012; Maresca et al., 2013). These CAIs discovered after the inorganic anion trithiocarbonate (CS_3_^2–^, TTC) have been evaluated by using kinetic and X-ray crystallographic studies, for understanding the binding of this relatively weak inhibitor to the human isoform hCA II (Innocenti et al., 2010). Afterward, it has been demonstrated that, due to the fact that both DTCs, similar to TTC, incorporate the CS_2_^–^ fragment, they bind through one of the sulfur atoms to the Zn^2+^ ion from the CA active site, interacting also with the conserved residues mentioned above in all α-CAs, Thr199, and Glu106. DTCs act as micromolar—low nanomolar CAIs against many isoforms, since their organic scaffold was observed to participate in various interactions with the CA active site (Adak et al., 2010; Supuran, 2012; McKenna and Supuran, 2014).

#### Carboxylic Acids

**FIGURE 2:**
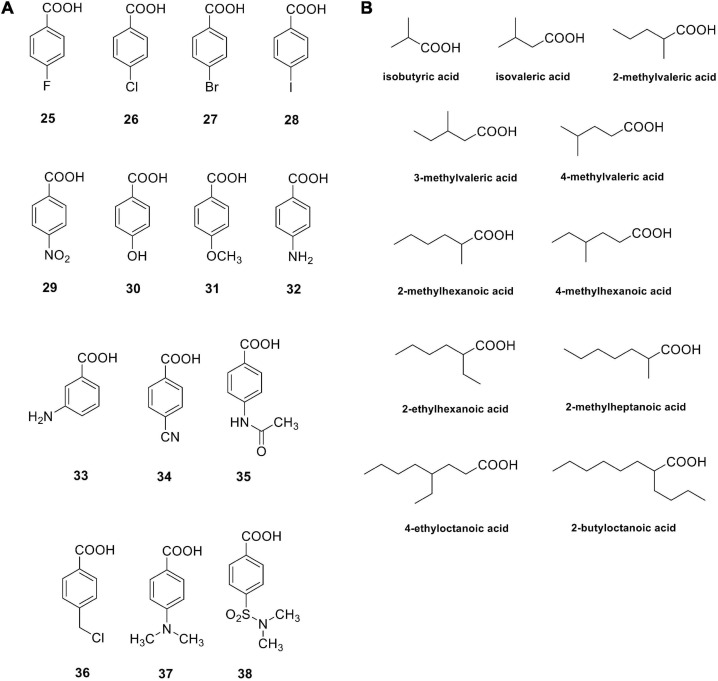
Carboxylic acids investigated as bacterial CAIs. Aromatic carboxylic acids **25–38 (A)**; aliphatic carboxylic acids **(B)**.

Carboxylic acids are a group of non-classical CA inhibitors, which include among others phenols, polyamines, fullerenes, coumarins and their derivatives (Lomelino et al., 2016b). Aromatic carboxylic acids (e.g., compounds **25**–**38** in Figure 2) as well as aliphatic such derivatives (Figure 2B) inhibit CAs by various mechanisms; they can coordinate the catalytic ion cofactor as anions in a mono- or bidentate manner, or can anchor to the Zn^2+^-coordinated water. Carboxylates may have thus access to the catalytic zinc displacing the bound water/hydroxide or impairing its catalytic effectiveness due to anchoring to it. This binding is similar to that observed for phenol-based or polyamine CAIs, which has been documented by X-ray crystallography (Lomelino et al., 2016b).

## *Helicobacter pylori* and Its Adaptation in the Stomach

In 1979, examining the tissue samples from patients subjected to a gastric biopsy, the pathologist J. Robin Warren noted many spiral-shaped curved bacteria below the thick mucus layer, covering the stomach inner wall. Later, Warren associated this infection with chronic superficial gastritis, and the nested bacteria were identified as belonging to the genus *Helicobacter* (Warren, 2000, 2006). Today, we know that a high percentage of people infected with *H. pylori* have superficial chronic gastritis. Besides, if left untreated, both *H. pylori* infection and inflammation can persist for decades, and sometimes even for all the lifetime (Rahman et al., 2020). Over the years, it has learnt that *H. pylori* causes a chronic inflammatory process, the peptic ulcer in the stomach and duodenum, the portion of the small intestine that originates from the pylorus (Zhu et al., 2020). The infection, previously considered to be of metabolic origin, strongly increases the risk of neoplasms, such as adenocarcinomas and lymphomas. For example, it was discovered that some varieties of *H. pylori* have a 40 kb DNA insertion element called cag pathogenicity island (cag PAI), containing about 32 genes encoding the bacterial type IV secretion system (Noto and Peak, 2012). The cag system enables the transmission of bacterial effector molecules into the gastric epithelial cells of the host. Some *H. pylori* strains slowly inject into the gastric cells one of the virulent proteins, called CagA that can trigger severe gastritis atrophy, dysplasia, and gastric adenocarcinoma, when a comparison with counterpart strains lacking this component was done (Amieva et al., 2003; Ye et al., 2003).

*Helicobacter pylori* is a Gram-negative pathogenic neutralophilic bacterium with a metabolism harmonized for a neutral pH development, but it is adapted to live in the overly acidic gastrointestinal environment (Tarsia et al., 2018). *H. pylori* genome encodes for α- and β-CAs. The α-CA (hpβCA) has a periplasmic localization, while the β-CA (hpβCA) is localized in the cytoplasm. It was aforementioned that the activity of *H. pylori* CAs could be an additional adaptation of the bacterium in the high acidic gastrointestinal environment (pH in the range 1–2). Urease and CAs are the two enzymatic systems used by the microbe for growing in this extreme environment (Capasso and Supuran, 2015a). These enzymes regulate the bacterial pH value determining an increase in the cytoplasm through ammonia production (NH_3_). Urea goes into the cytoplasm through the urea channel under acidic conditions, where the urease converts it into NH_3_ and CO_2_. In the cytoplasm, resulting CO_2_ is then hydrated by β-CA, while the periplasmic α-CA hydrates the CO_2_ diffused in the periplasm (Capasso and Supuran, 2015b). The produced ions (H^+^) by the CA-catalyzed reaction are used by NH_3_ to form NH_4_^+^ in the periplasm and cytoplasm, which neutralizes the entering acid in the above environments (Morishita et al., 2008). The combined action of urease and CAs result in the acid acclimatization of the pathogen within the stomach.

## Enzyme Activity and *in vitro* and *in vivo* Inhibition of the *Helicobacter pylori* Cas

### Enzyme Activity

Periplasmic hpβCA and cytoplasmic hpβCA are catalytically efficient for the CO_2_ hydration reaction with a k_*cat*_ values in the order of 10^5^ s^–1^. This catalytic constant is quite close to the k_*cat*_ of human isoenzyme hCA I (Nishimori et al., 2008).

### Inhibition by Substituted Benzene-Sulfonamides and Clinically Licensed Drugs

hpβCA and hpβCA were strongly inhibited by many sulfonamides/sulfamates **1–24** and **AAZ-HCT** (see Table 1) as well as by novel derivatives obtained by attaching 4-*tert*-butyl-phenylcarboxamido/sulfonamide tails to benzenesulfonamide/1,3,4-thiadiazole-2-sulfonamide scaffolds (Modak et al., 2015, 2016). Dorzolamide and simple 4-substituted benzenesulfonamides were feeble inhibitors (*K*_Is_ 873–4,360 nM). Sulfanilamide, orthanilamide, some of their derivatives, and indisulam showed a more strong inhibitory effect (*K*_Is_ 413–640 nM), whereas methazolamide, ethoxzolamide, dichlorophenamide, brinzolamide, topiramate, zonisamide, and others, worked as inhibitors of medium strength (*K*_Is_ 105–378 nM) (Nishimori et al., 2007; Table 1). For example, hpβCA was selectivity inhibited over the hCAII by acetazolamide, 4-amino-6-chloro-1,3-benzenedisulfonamide, 4-(2-amino-pyrimidin-4-yl)-benzenesulfonamide (K_Is_ in the range of 20–96 nM), and compounds incorporating lipophilic tails (*K*_Is_ = 12–84 nM) (Nishimori et al., 2006). Intriguingly, the hydrophilic pocket of hpβCA resulted more open with respect to that of hCA II. As a consequence, famotidine (**FAM**), an antiulcer drug incorporating a sulfamide, resulted in an excellent inhibition of hpβCA (Angeli et al., 2018; Table 1).

**TABLE 1:** Inhibition of human hCA I and hCA II isoforms as well as of *H. pylori* CAs (hpαCA and hpβCA) with sulfonamides **1–24** and the clinically used drugs **AAZ-FAM**.

Inhibitor	*K*_*I*_^*c*^ (nM)			
	hCA I^a^	hCA II^a^	hpαCA^a^	hpβCA^b^
**1**	45,400	295	426	16,400
**2**	25,000	240	454	1,845
**3**	28,000	300	316	8,650
**4**	78,500	320	430	2,470
**5**	25,000	170	873	2,360
**6**	21,000	160	1,150	3,500
**7**	8,300	60	1,230	1,359
**8**	9,800	110	378	1,463
**9**	6,500	40	452	1,235
**10**	6,000	70	510	1,146
**11**	5,800	63	412	973
**12**	8,400	75	49	640
**13**	8,600	60	323	2,590
**14**	9,300	19	549	768
**15**	6	2	268	64
**16**	164	46	131	87
**17**	185	50	114	71
**18**	109	33	84	38
**19**	95	30	207	39
**20**	690	12	105	37
**21**	55	80	876	236
**22**	21,000	125	1,134	218
**23**	23,000	133	1,052	450
**24**	24,000	125	541	15,250
**AAZ**	250	12	21	40
**MZA**	50	14	225	176
**EZA**	25	8	193	33
**DCP**	1,200	38	378	105
**DZA**	50,000	9	4,360	73
**BRZ**	45,000	3	210	128
**BZA**	15	9	315	54
**TPM**	250	10	172	32
**ZNS**	56	35	231	254
**SLP**	1,200	40	204	35
**IND**	31	15	413	143
**FAM**	922	58	21	50

*^a^Human/bacterial recombinant isozymes and stopped-flow CO_2_ hydrase assay method, as reported in Nishimori et al. (2008). ^b^Recombinant hpCA and stopped-flow CO_2_ hydrase assay method, as reported in this work, mean ± SE (from three different assays) and in Nishimori et al. (2007). ^c^Errors in the range of 5–10% of the shown data, as resulting from three different assays.*

### *In vivo* CA Inhibition

The involvement of CAs in the acid acclimation of *H. pylori* in the human stomach has been documented by administering CAIs, which inhibited the acid-producing machinery within the gut (Supuran, 2008). For example, acetazolamide was administrated in 1960 to treat American patients affected by peptic ulcers before the modern anti-ulcer agents were available (Buzas and Supuran, 2016). In 1968, Puscas administrated acetazolamide (2–4 g/day) to many ulcer patients obtaining considerable success even if the treatments were associated with a range of side effects. Recently, it has been shown that acetazolamide administration (500 mg) to volunteers with active *H. pylori* infection reduced the ability of *H. pylori* to adapt/survive in the acid environment of the stomach (Shahidzadeh et al., 2005). Other than acetazolamide, ethoxzolamide (**EZA**) can be considered a potential drug for developing new anti-*H. pylori* inhibitors since it kills the bacterium in cell cultures (Modak et al., 2019). Besides, EZA resistance did not develop easily in the *H. pylori* strains (P12, SS1,m and 26695) used for the experiments, and the compound seems to target multiple pathways since resistance acquisition was due to mutations associated with other genes than CAs (Rahman et al., 2020). In this context, we stress the fact that, recently, it has been demonstrated that the well documented vancomycin-resistant enterococci (VRE) might be addressed by targeting the *Enterococcus* CAs using a modified scaffold of acetazolamide (an inhibitor of the carbonic anhydrases) (Kaur et al., 2020). As a result, the authors identified two lead compounds having improved potency against clinical VRE strains (MIC from 0.007 to 1 μg/mL) (117). It is readily apparent that these results support the proof-of-concept that CAIs can be considered as novel antibacterials.

## *H.pylori* Outer Membrane Vesicles

The bacterial extracellular vesicles (EVs) are generated in a budding manner similar to that of the yeasts (Kim et al., 2015). Gram-negative bacteria, differently from the Gram-positive bacteria, produce extracellular vesicles by pinching off the outer membrane and, for this reason, are defined with the acronym OMVs (Outer Membrane Vesicles) (Liu et al., 2018). Distinctive features of OMVs are the lipopolysaccharide (LPS) and encapsulate periplasmic components, which are absent in the Gram-positive EVs. The vesicles generated by Gram-positive bacteria could bring inside various molecules, including nucleic acids, proteins, lipids, viruses, enzymes, and toxins. Depending molecules contained inside, these vesicles can have variegated roles. For example, they are involved in horizontal gene transfer, antibiotic resistance, microbial survival, microbial competition, nutrient acquisition, health benefits for the host, microbial virulence, cell-cell communication among bacteria and hosts, and biofilm formation (Ronci et al., 2019). The *H. pylori* OMVs are implicated in biofilm formation, and the presence of DNA inside these vesicles appears to be involved in “joining” OMV–OMV and OMV–cell communications (Grande et al., 2012, 2015). As aforementioned, Ronci et al. used mass spectrometry to identify periplasmic β-CA in the *H. pylori* OMVs generated *in vitro* from the microbe both in its planktonic and biofilm phenotypes (Ronci et al., 2019). Besides, β-CA hydratase activity was determined using the protonography, a technique selective for the detection of CAs. As a result, it was demonstrated that the amount of the periplasmic β-CA was higher in the planktonic OMVs (pOMVs) than in the biofilm OMVs (bOMVs). Furthermore, it was observed that the content of β-CA increased in pOMVs over time.

Moreover, the biofilm phenotype, a complex structure in which bacteria adhere to a surface and are embedded in a self-produced EPS (extracellular polymeric substance) matrix, is a condition used by pathogenic bacteria to improve their survival, bacterial infection, and resistance to the effects of antimicrobial agents (Parsek and Singh, 2003; Grande et al., 2014). *H. pylori* tends to form a biofilm on human gastric mucosa (Yonezawa et al., 2015), and biofilm cells are more resistant to the effects of antimicrobial agents (Carron et al., 2006). Generally, the first-line therapy to eradicate *H. pylori* infection is based on a combination of drugs, such as proton pump inhibitor (PPIs), amoxicillin, clarithromycin (CAM) or metronidazole, and fluoroquinolones (Bang et al., 2020). Novel approaches to prevent biofilm formation and to treat infections by biofilm-forming bacteria are currently under development (Bjarnsholt et al., 2018). The identification of periplasmic β-CA in pOMVs and its specific inhibition with the classical CAIs might shed new light on this enzyme’s role in the *H. pylori* colonization, survival, persistence, and pathogenesis.

## CA Inhibitors in Combination With Probiotic Strains

In the literature, it has been reported the possible existence of a correlation between intestinal microbiota (i.e., microbial populations living in the intestine) and various autoimmune diseases, such as systemic lupus erythematosus or autoimmune liver diseases (Manfredo Vieira et al., 2018). Kriegel and colleagues noted that the bacterium *Enterococcus gallinarum*, a very rare enterococcus, has been often identified in the intestinal flora and liver of patients with lupus; thus, it was considered as a trigger of the systemic lupus erythematosus (Manfredo Vieira et al., 2018). In experiments on mice, it was demonstrated that these bacteria can overcome the small intestine barrier and quickly reach the liver and other organs, particularly the spleen and lymph nodes. In this way, the bacterium triggers an inflammatory process, which allows the secretion of chemical messengers equal to those observed in subjects with lupus, inducing the proliferation of autoantibodies that also attack the cells of the organism. In contrast, some bacterial strains, which live within the microbiota, function as brakes against intestinal tumors (Zagato et al., 2020). For example, it has been observed the absence of *Holdemanella biformis*, a bacterium belonging to the family of Erysipelotrichaceae, in the microbiota of patients in an early stage of development of intestinal cancer. These bacteria have antitumor properties capable of blocking the uncontrolled proliferation of cells, which happens in the case of a lack of them in the gut (Zagato et al., 2020). Thus, it is reasonable to believe that these anti-tumorigenic bacteria have a strong diagnostic, therapeutic, and translational potential.

In *H. pylori*-infected individuals, the gastric microbiota is similar to that of the non-infected persons. In general, infected persons have 52.6% of Proteobacteria, 26.4% of Firmicutes, 12% of Bacteroidetes and 6.4% of Actinobacteria (Llorca et al., 2017; Ozbey et al., 2020). The resident gastric microflora may interfere with *H. pylori*’s proliferation and gut disease. For this reason, the pharmacological treatments for eradicating *H. pylori* from the gastric mucosa can be ameliorate using probiotics (Emara et al., 2014; Maccelli et al., 2020). The effect of new antimicrobial molecules is rarely evaluated; thus, the identification of new drugs that possess a selective toxicity between pathogens and some components of the human microbiota might represent an important step in the clinical field (Grande et al., 2020). Therefore, the identification of probiotic strains, which do not possess the CAs and constitute a significant component of the human microbiota (Martin et al., 2013) used in combination with innovative drugs, such as those coming from the modified scaffold of CAIs, might represent an innovative anti-*H. pylori* treatment. Not being affected by the inhibitor, the probiotic can exert a synergistic effect improving the antimicrobial action of the (bactericidal or bacteriostatic) CA inhibitors. Besides, the probiotic, by educating or stimulating the host immune system, could also contribute to the efficacy of the CA inhibitor. Thus, this combination might represent an innovative and successful strategy to fight infections without altering the normal microbiota.

## Conclusion

At least four classes of CAs (α, β, γ, and ι) are present in Bacteria. During growth, microbes require CO_2_ and HCO_3_^–^ to support their metabolism, as well as H^+^ ions/bicarbonate for the balance of the pH value. CAs with their activity correctly balance the interconversion of inorganic these species. Thus, CAs play essential roles in the life cycle of pathogenic and non-pathogenic bacteria, and their inhibition prejudices the growth of the microbe. This paves the way for designing novel anti-infective drugs, which function differently from the standard antibiotics. The involvement of CAs in the lifecycle, pathogenicity, and virulence of several species (e.g., *H. pylori*, *V. cholerae*, *B. suis*, *S. enterica*, *P. aeruginosa*, and *Enterococcus* spp.) of human pathogens is not new, but only recently programs to develop agents that specifically and selectively inhibit these enzymes have been initiated. Presently, many bacterial species have been investigated for the presence of CAs belonging to all four classes mentioned above. Furthermore, many bacterial CAs have been prepared as recombinant enzymes and thoroughly characterized by a biochemical viewpoint and for their ability to be inhibited by various compounds. These enzymes were effectively inhibited by the classical CAIs, such as the sulfonamides and their derivatives, sulfamates, sulfamides, (in)organic anions, and some of them by dithiocarbamates as well as carboxylic acids. More exciting is the discovery that ethoxzolamide can kill *H. pylori in vitro* and *in vivo*, and that the bacterial resistance to this compound does not develop easily. The recent study on the efficacy of acetazolamide and some of its derivatives to act as inhibitors of vancomycin resistant enterococci (VRE) is a breakthrough in the field (Kaur et al., 2020). The drug design campaign reported in the same study led to the identification of sulfonamide derivatives, which seem to be orders of magnitude more efficient against VRE, when compared to the clinically used agent linezolid. Drug design campaigns were also useful in finding *H. pylori*-selective CAIs belonging to the sulfonamide class. Thus, it is not impossible to hypothesize that the field of CAIs as anti-infectives may lead to relevant developments in the near future and future dedicated studies are necessary in this context.

## Author Contributions

All authors contributed to the article and approved the submitted version.

## Conflict of Interest

RG and SC have a scientific collaboration with BioGaia AB (Stockholm, Sweden). The remaining authors declare that the research was conducted in the absence of any commercial or financial relationships that could be construed as a potential conflict of interest.
